# Traditional Chinese Medicine *Xuebijing* Treatment Is Associated with Decreased Mortality Risk of Patients with Moderate Paraquat Poisoning

**DOI:** 10.1371/journal.pone.0123504

**Published:** 2015-04-29

**Authors:** Ping Gong, Zhidan Lu, Jing Xing, Na Wang, Yu Zhang

**Affiliations:** Emergency Department, First Affiliated Hospital of Dalian Medical University, Dalian, 116011, Liaoning province, People’s Republic of China; The Ohio State University, UNITED STATES

## Abstract

Paraquat poisoning causes multiple organ injury and high mortality due to severe toxicity and lack of effective treatment. *Xuebijing* (XBJ) injection, a traditional Chinese medicine preparation of five Chinese herbs (Radix Salviae Miltiorrhiae, Rhizoma Chuanxiong, Flos Carthami, Angelica Sinensis and Radix Paeoniae Rubra), has an anti-inflammatory effect and is widely used in the treatment of sepsis. This retrospective study was designed to evaluate the effects of XBJ combined with conventional therapy on mortality risk of patients with acute paraquat poisoning. Out of 68 patients, 27 were treated with conventional therapy (control group) and 41 were treated with intravenous administration of XBJ (100 ml, twice a day, up to 7 days) plus conventional therapy (XBJ group). Vital organ function, survival time within 28 days and adverse events during the treatment were reviewed. Results indicated that XBJ treatment significantly increased median survival time among patients ingesting 10-30 ml of paraquat (*P*=0.02) compared with the control group. After adjustment for covariates, XBJ treatment was associated significantly with a lower mortality risk (adjusted HR 0.242, 95% CI 0.113 to 0.516, *P*=0.001) compared with the control group. Additionally, compared with Day 1, on Day 3 the value of PaO_2_/FiO_2_ was significantly decreased, and the values of serum alanine aminotransferase, creatinine and troponin T were significantly increased in the control group (all *P*<0.05), but these values were significant improved in the XBJ group (all *P*<0.05). Only one patient had skin rash with itch within 30 minutes after injection and no severe adverse events were found in the XBJ group. In conclusion, XBJ treatment is associated with decreased mortality risk of patients with moderate paraquat poisoning, which may be attributed to improved function of vital organs with no severe adverse events.

## Introduction

Paraquat is a widely used herbicide in agriculture. There are numerous cases of paraquat poisoning by oral ingestion either accidentally or intentionally. The mortality rate of paraquat poisoning ranges from 50% to 90%, with no specific anticdote [1]. Clinical manifestations and outcomes of acute paraquat poisoning depend on the volume of paraquat ingested orally. A volume of 20 ml of 20% liquid paraquat (w/v) is likely to cause death within 1–4 days, while smaller quantities (<20 ml) may cause irreversible lung fibrosis and renal failure, leading to death within several weeks [2]. Patients are often treated aggressively with a conventional therapy including gastric lavage, fluid infusion, antioxidants, cyclophosphamide, corticosteroids and haemoperfusion [3]. Recently, extracorporeal elimination such as haemoperfusion has been used clinically in treating acute paraquat poisoning despite its controversial effects [4,5]. However, the outcome of paraquat poisoning is still disappointing, with a high mortality [6].

**Fig 1:**
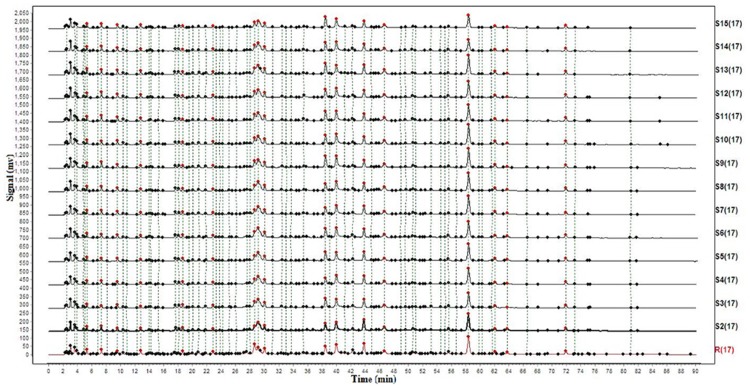
Fingerprint chromatograph of XBJ.

*Xuebijing* (XBJ) injection, a traditional Chinese medicine preparation, is widely used in the treatment of sepsis in China; the possible mechanisms are believed to be associated with antagonizing pro-inflammatory factors [7–9]. XBJ is extracted from five Chinese herbs including Radix Salviae Miltiorrhiae, Rhizoma Chuanxiong, Flos Carthami, Angelica Sinensis and Radix Paeoniae Rubra; at least 21 compounds have been found in XBJ, including amino acids, phenolic acids, flavonoid glycoside, terpene glycoside, and phthalide [8,10]. Radix Salviae Miltiorrhiae [11–15], Rhizoma Chuanxiong [16,17], Flos Carthami [18,19] and Angelica Sinensis [20–22] are known to exert anti-inflammatory effects. The quality of XBJ has been strictly controlled according to the standards of the China Ministry of Public Health, and fingerprint technology is adopted in the production as a quality control measure (Fig 1), so XBJ quality does not vary substantially from batch to batch or from laboratory to laboratory. Recently, XBJ has been used in patients with acute paraquat poisoning in China [23,24]. Zheng et al have reported that the treatment with XBJ plus conventional therapy significantly decreases transforming growth factor (TGF)-beta I and procollagen type III peptide (PIIIP) levels in patients with paraquat poisoning [24]. However, the clinical therapeutic efficacy of XBJ in patients with acute paraquat poisoning has not been well established. In the present study, we retrospectively reviewed laboratory and clinical data to determine the effects of XBJ in combination with conventional therapy on vital organ function and the survival of patients with acute paraquat poisoning.

## Materials and Methods

### Ethics statement

This study was conducted in accordance with the Declaration of Helsinki. The study protocol, the use of XBJ in patients with acute paraquat poisoning and clinical data collection and review were approved by the Medical Ethics Committee of the First Affiliated Hospital of Dalian Medical University, Dalian, China. Since this study only involved retrospective review of the existing data, the Institutional Review Board approval was obtained with a waiver for specific informed consent from the patients. However, the informed consent regarding the risk of acute paraquat poisoning and treatments including haemoperfusion and administration of XBJ, cyclophosphamide and corticosteroids were obtained from each of the patients upon their initial admission. In addition, there were no identified risks to the patients of this study since the data were analyzed anonymously.

### Patients

We retrospectively reviewed the medical records of 68 patients with acute paraquat poisoning admitted to the Emergency Intensive Care Unit from June 1, 2008 to May 31, 2013. All patients included in the present study arrived at the emergency department within 24 hours of oral ingestion of paraquat. Acute paraquat poisoning was diagnosed based on history, clinical signs for typical paraquat poisoning such as mouth lesions and/or blue colouration around the mouth, and laboratory examinations (especially urine paraquat test). The positive results of the urine paraquat tests were resulted from paraquat reduction by sodium dithionate in an alkaloid environment, which results in the production of blue radicals [25]. Navy blue or dark blue color usually indicates significant paraquat poisoning [25].

### Inclusion and exclusion criteria

Patients were included in the present study if they were older than 18 years of age and had positive urine paraquat tests. Patients were excluded if exposed by dermal [26] or intravascular [27] routes or without detectable paraquat levels in their urine, or had major comorbidities, such as heart, lung, renal, or liver diseases, shock or cancer. Determination of major comorbidities was based on medical records of clinical and laboratory examinations.

### Treatment

Out of the 68 patients, 27 were treated with conventional therapy (control group) and 41 were treated with intravenous XBJ administration (100 ml, twice a day, up to 7 days) plus the conventional therapy (XBJ group). This conventional therapy included gastric lavage, fluid infusion, antioxidants (vitamin C 50 mg/kg/day and reduced glutathione sodium 50 mg/kg/day), cyclophosphamide (15 mg/kg/day, up to 2 days),corticosteroids (methylprednisolone, 15 mg/kg/day, up to 3 days) and emergency haemoperfusion. Emergency haemoperfusion was performed on all of the patients through a double lumen femoral venous catheter (Gambro, Hechingen, German) for 2 hours a day, up to 5 days, at a blood flow rate of 200 ml/min, using AK-200 haemoperfusion machine (Gambro, Hechingen, German) and a resin-containing column coated with polycarbonate (HA-230, Zhu Hai Jian Fan, Zhu Hai City, China). No oxygen therapy was administered to patients with dyspnea, but ventilation with a fraction of inspiration O_2_ (FiO_2_) as low as possible (21%-30%) was considered if arterial oxygen saturation was lower than 70% or arterial partial pressure of oxygen (PaO_2_) was lower than 50 mmHg.

### Variables

The following variables were collected and reviewed for each patient: sex, age, paraquat ingestion volume (based on the remaining amounts of paraquat in the bottles provided by companions of patients upon their initial admission and the description of patients and their companions about the amount of orally ingested paraquat also were considered as a reference), completed blood cell counts and coagulation (XT 4000i, Sysmex America, Lincolnshire, Illinois, USA), routine urine analysis (Uf500i, Sysmex America, Lincolnshire, Illinois, USA), electrolytes, liver and renal functions (V5600, Johnson & Johnson, New Brunswick, New Jersey, USA), troponin T (ADVIA Centaur CP, Siemens, Munich, German) and arterial blood gas (Rapidpoint 405, Siemens, Munich, German) measured on Days 1, 3, and 7 following admission, and Acute Physiology and Chronic Health Evaluation II (APACHE II) scores [28] assessed on admission. Survival time within 28 days was obtained. In addition, adverse events during the treatment were reviewed. Adverse events and severe adverse events were defined according to the definitions of the quality control standard for drug clinical trials issued by the China Food and Drug Administration [29].

### Statistical analyses

Continuous variables are presented as mean ± standard deviation (SD) except for age, BMI and time to initial haemoperfusion, which are expressed as median and range. A Student’s t test was used to compare the means of continuous variables with normally distributed data. Otherwise, a Mann—Whitney U test was used for non-normally distributed data. The 28-day survival rates were compared using Pearson Chi-Square or Fisher’s exact test. Cumulative survival curves as a function of time were generated using the Kaplan—Meier method and compared by log-rank test. Cox proportional hazard models were then used to assess the impact of covariates (age, gender, body mass index, time to gastric lavage, APACHE II scores, volume of oral paraquat, and time to haemoperfusion) on mortality. Estimated risks of death were reported as hazard ratios (HR) with 95% confidence intervals (CI). PaO_2_/FiO_2_, serum alanine aminotransferase (ALT), creatinine and troponin T (TnT) were compared using a repeated-measures ANOVA including the above covariates, followed by the Bonferroni test for multiple comparisons. Differences were considered statistically significant when *P* was <0.05. The data were analyzed using the software package SPSS 16.0 (SPSS Inc., Chicago, Illinois, USA).

## Results

### Patients’ characteristics

At baseline, no group differences were observed for age, gender, BMI or APACHE II scores (all *P*>0.05, Table 1). There were no significant differences in the median time to gastric lavage, the volumes of oral paraquat ingestion and the median time to initiate haemoperfusion between the control and XBJ groups (all *P*>0.05, Table 1). Most patients (95.6% of all cases) ingested paraquat deliberately due to suicide attempt. Sixty eight patients were further stratified according to the volumes of oral 20% liquid paraquat (w/v), as indicated in Table 2.

**Table 1:** Baseline characteristics.

	XBJ	Control	*P* value
**Age** (years)			
**<10 ml**	33 (30, 38)	35 (31, 39)	NS
**10–30 ml**	32 (25, 42)	34 (28, 41)	NS
**>30 ml**	34 (32, 43)	36 (30, 45)	NS
**Female** (%)			
**<10 ml**	6/9(66.7%)	4/6 (66.7%)	NS
**10–30 ml**	18/22 (81.8%)	12/14 (85.7%)	NS
**>30 ml**	8/10 (80.0%)	6/7 (85.7%)	NS
**BMI** (kg/m^2^)			
**<10 ml**	22.7 (16.8, 31.1)	21.5 (17.5, 24.3)	NS
**10–30 ml**	23.0 (15.1, 33.2)	21.2 (13.2, 31.6)	NS
**>30 ml**	21.7 (15.8, 30.2)	22.1 (15.5, 33.2)	NS
**Time to gastric lavage** (hours)			
**<10 ml**	2.1±0.7	2.0±0.4	NS
**10–30 ml**	2.1±0.5	2.0±0.7	NS
**>30 ml**	2.1±0.3	2.0±0.5	NS
**APACHE II scores**			
**<10 ml**	5±1	5±1	NS
**10–30 ml**	8±1	7±2	NS
**>30 ml**	10±2	10±2	NS
**Volume of oral paraquat** (ml)			
**<10 ml**	6.2±1.6	6.5 ±1.9	NS
**10–30 ml**	18.0±5.0	18.0 ±3.6	NS
**>30 ml**	42.3±8.0	43.3 ±5.2	NS
**Time to haemoperfusion**(hours)			
**<10 ml**	5.0 (3.0, 8.5)	5.8 (3.5, 9.0)	NS
**10–30 ml**	5.6 (2.8, 10.0)	5.5 (3.0, 10.0)	NS
**>30 ml**	5.8 (3.5, 9.5)	5.5 (2.5, 8.0)	NS

Note: XBJ, *Xuebijing* injection; BMI, Body mass index; APACHE II, Acute Physiology and Chronic Health Evaluation II; NS, no significance.

**Table 2:** 28-day survival rates of patients ingesting different volumes of 20% liquid paraquat (w/v).

	<10 ml	10–30 ml	>30 ml
**XBJ**	100% (9/9)	40.9% (9/22)	20.0% (2/10)
**Control**	100% (6/6)	21.4% (3/14)	0% (0/7)

Note: XBJ, *Xuebijing* injection.

### Effect of XBJ on survival rates of patients with paraquat poisoning

**Fig 2:**
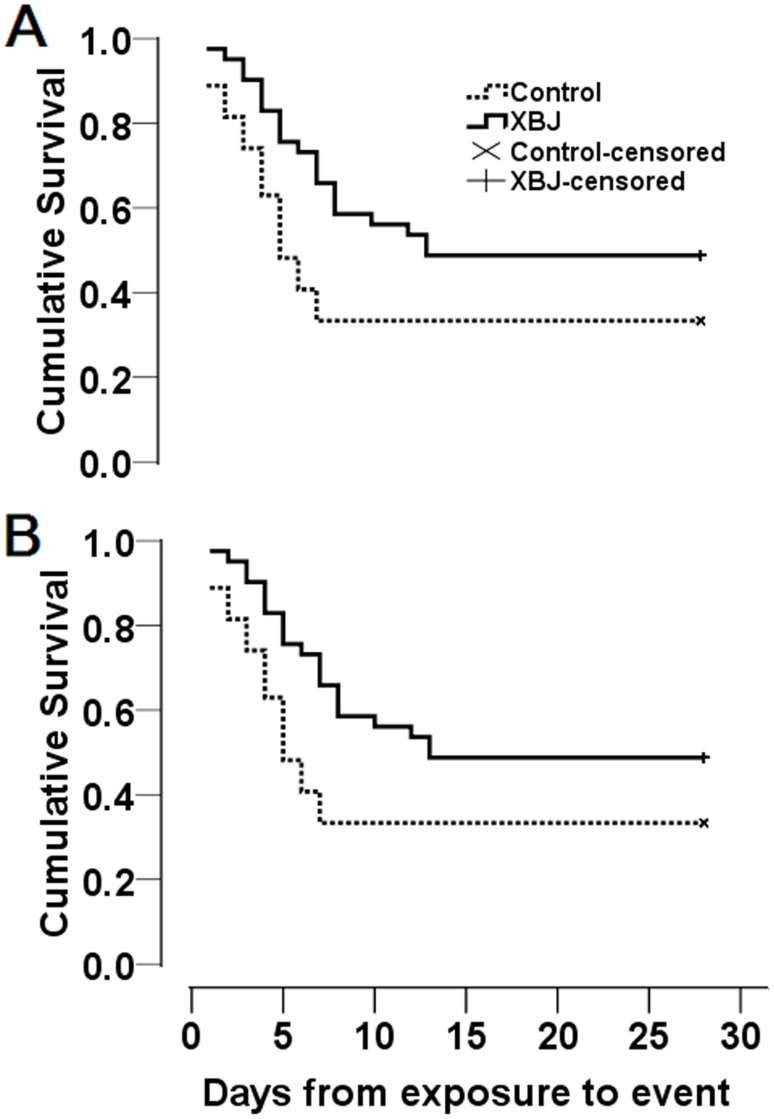
Kaplan-Meier Survival Curves for (A) all patients and (B) patients ingesting 10–30 ml of 20% liquid paraquat (w/v). The differences in survival curves between the control and XBJ groups were not significant in A (*P* = 0.06), but significant in B (*P* = 0.02), as compared using a Log rank test. XBJ, *Xuebijing* injection.

Patients treated with XBJ had a slight increase in 28-day survival rate, compared with their counterparts in the control group, but this difference (15.5%) did not reach statistical significance (48.8% *vs*.33.3%, *P* = 0.21, Fig 2A). Similarly, the Kaplan—Meier survival curves (Fig 2A) showed that median survival time was slightly increased from 5.0 days (95% CI 3.3–6.7 days) with conventional therapy to 13 days (95% CI 6.5–18.6 days) with XBJ treatment plus conventional therapy, however Log rank tests indicated that there was no significant difference between the two survival curves (*P* = 0.06).

Interestingly, following stratification based on the volume of oral ingestion, patients ingesting 10–30 ml paraquat with XBJ treatment showed a significantly increased median survival time when compared to patients in the control group [12.0 days (95% CI 6.3–17.7 days) *vs* 5.0 days (95% CI 3.8–6.2 days), Fig 2B]. The Log rank test indicated a significant difference between the two survival curves (*P* = 0.02). For patients ingesting 10–30 ml of paraquat and treated with XBJ, an increase in 28-day survival rate (19.5%) was observed, compared with patients in the control group (40.9% *vs* 21.4%, Table 2), however the difference did not reach a significant level (*P* = 0.29). After adjustment for covariates including age, gender, body mass index, time to gastric lavage, APACHE II scores, volume of oral paraquat, and time to haemoperfusion, XBJ treatment was associated significantly with a lower mortality risk (adjusted HR 0.242, 95% CI 0.113 to 0.516, *P* = 0.001; Table 3) compared with the control group. Other significant risk factors for mortality among patient with paraquat poisoning included volume of oral paraquat (adjusted HR 1.061, 95% CI 1.032 to 1.090, *P* = 0.001; Table 3) and APACHE II scores (adjusted HR 1.283, 95% CI 1.064 to 1.547, *P* = 0.009; Table 3). In addition, we found that patients ingesting less than 10 ml of paraquat all survived 28 days (Table 2), and patients ingesting more than 30 ml of paraquat died within the initial several days, except for two patients who survived 28 days in the XBJ group (Table 2).

**Table 3:** Significant risk factors for mortality among patients with paraquat poisoning.

Variables	Adjusted Hazard ratio (95% CI)	*P* value
Group	0.242 (0.113 to 0.516)	0.001
Age	0.964 (0.885 to 1.050)	0.404
Female	1.006 (0.383 to 2.645)	0.990
BMI	1.014 (0.941 to 1.091)	0.721
Time to gastric lavage	1.204 (0.627 to 2.315)	0.577
APACHE II scores	1.283 (1.064 to 1.547)	0.009
Volume of oral paraquat	1.061 (1.032 to 1.090)	0.001
Time to haemoperfusion	1.079 (0.892 to 1.306)	0.434

Note: Adjusted for covariates including age, gender, body mass index, time to gastric lavage, APACHE II scores, volume of oral paraquat, and time to haemoperfusion. APACHE II, Acute Physiology and Chronic Health Evaluation II; BMI, body mass index; CI, confidence interval.

### Effects of XBJ on vital organ function following paraquat poisoning

On admission (Day 1), the values of oxygenation index (PaO_2_/FiO_2_), serum alanine aminotransferase (ALT), creatinine and troponin T (TnT) were similar in the XBJ and control groups (all *P*>0.05, Table 4). Compared with Day 1, on Day 3 the value of PaO_2_/FiO_2_ was significantly decreased, indicating the deterioration of lung function, and the values of serum ALT, creatinine and TnT were significantly increased, indicating the deterioration of the functions of liver, kidney and heart, in the control group (all *P*<0.05, Table 4). However, these deteriorations were significant improved in the XBJ group (all *P*<0.05, Table 4). On Day 7, improved serum levels of ALT and creatinine were still observed in the XBJ group (both *P*<0.05, Table 4), but the PaO_2_/FiO_2_ and serum TnT levels were similar in the two groups.

**Table 4:** Vital organ functions in both groups during XBJ treatment.

	*n*	PaO_2_/FiO_2_	ALT (mmol/L)	Creatinine (μmol/ L)	TnT (μg/L)
**Day 1**					
**XBJ**	41	282±56	88±64	108.6 ±39.2	0.51 ±0.35
**Control**	27	279±62	92±64	115.6 ±39.4	0.58 ±0.37
**Day 3**					
**XBJ**	37	211±92*	257±207*	257.4 ±133.0*	1.07 ±0.87*
** Control**	20	155±108**	388±240**	374.0 ±152.8**	1.69 ±1.30**
**Day 7**					
**XBJ**	27	335±94	64±44*	161.0 ±131.8*	0.36 ±0.67
** Control**	9	331±20	109±81	272.0 ±102.8	0.38 ±0.26
***F***		3.535	6.675	8.747	6.119
***P***		0.036	0.005	0.001	0.020

Note: XBJ, *Xuebijing* injection; ALT, alanine aminotransferase; TnT, troponin T.

*P<0.05 XBJ group *vs*. control group;

**P<0.05 control group on Day 3 *vs*. control group on Day 1.

### Safety Evaluation

Only one patient had skin rash with itch within 30 minutes after injection and no severe adverse events were found in the XBJ group. However, the patient rapidly recovered from skin rash with itch due to acute allergy after injected with diphenhydramine (20 mg intramuscularly).

## Discussion

The key findings of the present study were that XBJ plus conventional therapy improved vital organ function of patients with acute paraquat poisoning, and increased median survival time of patients with moderate paraquat poisoning and decreased mortality risk. In this retrospective study, we found that XBJ did not have a statistically significant benefit in reducing all-cause mortality in patients with paraquat poisoning, which was consistent with the findings from a recent meta-analysis [30]. The meta-analysis was based on the findings of two small randomized controlled trials including 84 patients. However, we observed that, for patients with moderate paraquat poisoning (10–30 ml), XBJ was significantly beneficial in increasing median survival time and decreased mortality risk. We speculate that this significant benefit could be due to two reasons. First, it is well known that the clinical manifestations of paraquat poisoning depend upon the quantity ingested [6], as also supported by our finding that volume of oral paraquat was associated significantly with the mortality risk. Large amounts of paraquat ingested (>50–100 ml of 20% ion w/v) result in fulminant organ failures: pulmonary oedema, cardiac, renal and hepatic failure [6], followed by death within several hours to a few days [6,25]. In the present study, almost all of the patients ingesting more than 30 ml of paraquat died within the initial several days, except for two patients who survived 28 days following XBJ treatment. Additionally, all patients ingesting less than 10 ml of paraquat survived 28 days. Smaller amounts of paraquat usually lead to toxicity in the two key target organs (kidneys and lungs) developing within 2–6 days [6]. These patients could survive when treated with proper conventional therapy. Therefore, the survival benefits of XBJ could not be found in these patients ingesting less than 10 ml or more than 30 ml of paraquat.

Second, XBJ can alleviate paraquat-induced systemic inflammatory responses and secondary injury to multiple vital organs. Paraquat-induced toxicity is mediated through redox-cycling and subsequent generation of reactive oxygen species (ROS) [1,6,31–33], whereas activation of NF-kB from its dormant form [34] by ROS and subsequently induced inflammatory responses also contribute to paraquat-induced multiple organ injury [6,35–37]. Increasing evidence has documented that XBJ inhibits inflammatory responses and thereby protects against secondary multiple organ injury in many clinical scenarios such as severe sepsis [7,9,38] and heat stroke [39]. Therefore, this inhibition of XBJ to inflammatory responses may play a pivotal role in alleviating paraquat-induced multiple vital organ injury.

Adverse drug reactions/events associated with traditional Chinese medicine injections have been being focused on owing to their complex chemical ingredients, un-unified quality standard and other problems in production. Recently, Kong et al analyzed drug reactions/events of XBJ injection reported from 1994 to 2011 and found that 14 cases had different degrees of drug reactions in the seven years, most of which were due to non-standard use of XBJ and allergic condition of patients[40]. However, only one patient had skin rash with itch within 30 minutes during XBJ treatment and no severe adverse events were observed in the present study. It was mainly attributed to standard use and the safety of XBJ resulted from fingerprint technology as a strict quality control.

The present study has certain limitations. First, it was a retrospective analysis of patients with paraquat poisoning with a limited sample size. Second, we only analyzed the survival time within 28 days, but some patients could ultimately die of severe anoxia due to progressive fibrosis up to 5 weeks later [6]. Third, there were no data on the effects of XBJ on oxidative stress or inflammation or data on blood paraquat levels available for this analysis. Finally, the controls were selected from a historical group before XBJ was introduced and from patients who declined XBJ treatment due to cost (and therefore a likely to be less well off), and either of these might introduce a systematic difference in patient outcome.

In conclusion, XBJ treatment decreased mortality risk of patients with moderate paraquat poisoning, which may be attributed to improved function of vital organs with no severe adverse events.
