# Correction: Traditional Chinese medicine *Xuebijing* treatment is associated with decreased mortality risk of patients with moderate paraquat poisoning

**DOI:** 10.1371/journal.pone.0130508

**Published:** 2015-06-15

**Authors:** Ping Gong, Zhidan Lu, Jing Xing, Na Wang, Yu Zhang

The following information is missing from the Funding section: This study was supported by the Liaoning Province Science and Technology Plan Project (2012225020).
